# Personality Disorders and Personality Profiles in a Sample of Transgender Individuals Requesting Gender-Affirming Treatments

**DOI:** 10.3390/ijerph17051521

**Published:** 2020-02-27

**Authors:** Annalisa Anzani, Chiara De Panfilis, Cristiano Scandurra, Antonio Prunas

**Affiliations:** 1 Department of Psychology, University of Milano–Bicocca, 20126 Milan, Italy; antonio.prunas@unimib.it; 2 CREST, Centro per lo studio e la terapia dei disturbi di personalità, 20145 Milan, Italy; 3 Department of Medicine and Surgery, University of Parma, 43121 Parma, Italy; chiara.depanfilis@unipr.it; 4 Department of Neuroscience, Reproductive Sciences and Dentistry, University of Naples Federico II, 80131 Naples, Italy; cristiano.scandurra@unina.it

**Keywords:** personality disorders, maladaptive personality traits, alternative model, transgender, gender-affirming treatments

## Abstract

The study aims to explore the personality patterns of a group of transgender individuals who accessed an Italian gender clinic to undergo gender affirming treatments, by evaluating both dimensional personality domains proposed by the Alternative Model of Personality Disorders and categorical DSM-IV personality disorder (PD) diagnoses. Eighty-seven participants (40 transgender women and 47 transgender men) completed the Personality Inventory for DSM-5 and the Structured Clinical Interview for DSM-IV Axis II personality disorders. Scores obtained were compared to those of the normative samples of cisgender women and men. Results indicated that transgender women scored lower than cisgender women on two main domains (Negative Affectivity and Psychoticism) and on seven facets. As for transgender men, lower scores than cisgender men were found on Antagonism and on five facets. Transgender men scored higher than cisgender men on Depressivity. Nearly 50% of participants showed at least one PD diagnosis, with no gender differences in prevalence. Borderline PD was the most frequent diagnosis in the overall sample. Self-report measures provide a less maladaptive profile of personality functioning than the clinician-based categorical assessment. Results are interpreted in the light of the Minority Stress Model and support the need for a multi-method assessment of personality in medicalized transgender people.

## 1. Introduction

Mental health among transgender people is a sensitive topic and the role of mental health professionals in the diagnosis of gender dysphoria and the diagnosis itself are still controversial [1,2]. Evidence suggests that medicalized transgender individuals have a higher prevalence of psychopathology and personality disorders (PDs) than the general population (see Dhejne et al. [2] for a review). This datum should be framed within the minority stress theory (MST) [3,4], a theoretical framework assessing the role of psychosocial stressors in affecting health and well-being. Indeed, transgender people experience disproportionate rates of stressful life events due to their gender nonconformity or expression, and such stress may directly or indirectly (i.e., through the action of internalized stigma) affect their mental health and also cause major psychiatric disorders (i.e., mood disorders, anxiety, substance use disorders) [3,5,6,7].

Research on PD in medicalized transgender people is significant, as personality pathology might influence the clinical manifestations of gender dysphoria (GD) and the gender transition process. For instance, borderline PD was associated with younger age at the time of application to the gender clinic and tended to be related with greater GD severity [8]. Most importantly, a thorough assessment and understanding of the client’s personality functioning (in terms of identity stability, self-directedness, and interpersonal connections with others) is an important prognostic indicator that might help identify possible hurdles or concerns to be addressed in the gender transition process. As underlined in the *Standards of Care of the World Professional Association of Transgender Health 7th version* [9], mental health concerns (including PDs) “can be significant sources of distress and, if left untreated, can complicate the process of gender identity exploration and resolution of gender dysphoria” (p. 25). Therefore, within an affirmative and not pathologizing approach, mental health professionals should screen for both psychopathology and personality pathology during the assessment phase with GD clients, and take it into account when establishing the overall treatment plan. This allows to tailor the treatment to the patient’s specific needs, to help them navigate the transition process, to improve their quality of life and, ultimately, to reduce GD.

Notwithstanding these premises, no previous studies have explored the personality profiles of transgender people according to the new conceptual framework for PDs introduced by the *DSM-5 Alternative Model for Personality Disorders* (AMPD) [10]. Thus, the current study aimed at exploring the personality patterns of a sample of transgender individuals by evaluating both the dimensional personality domains proposed by the AMPD and the categorical DSM-IV PD diagnoses. In the following paragraphs, we will first provide an overview of research on psychopathology and PD in transgender individuals. We will then address the dimensional models of PDs. 

### 1.1. Personality Disorders in the Transgender Population

Transgender people with a diagnosis of GD and attending transgender healthcare services show higher rates of psychiatric disorders, and particularly anxiety and depressive disorders, than the cisgender population [4]. For example, both Smith et al. [11] and Jones [12] reported that transgender women (assigned male at birth) have a high likelihood of being diagnosed with depression. Jones et al. [13] and Bolger [14] reported similar results in transgender men (assigned female at birth). 

Some cross-sectional studies have also evaluated prevalence estimates of personality pathology in the transgender population [15,16,17,18,19,20,21,22], although research findings on this topic are still mixed and inconclusive. Overall, the prevalence of DSM-IV PDs in samples of medicalized transgender people spreads over a range between 4.3% [16] and 81.4% [21], with some studies [16] reporting a prevalence rate even inferior to what was found in large epidemiological samples [23]. Cluster B PDs, and particularly borderline and narcissistic PDs, were identified as the most frequently diagnosed Axis II disorders in these samples [18,19,20,21,24,25,26].

Other studies reported a high prevalence of Cluster C disorders [8], in particular avoidant PD [8,17], and of some specific Cluster A disorders, namely paranoid and schizoid PDs [8,15,26]. No differences seem to emerge when the prevalence of PDs is compared between people in the male and female spectrum [8,20], nor between medicalized transgender individuals with early and late onset [8].

In spite of its methodological limitations [2] and the relative scarcity of studies on this topic, research on PD prevalence in medicalized transgender people may have a twofold significance. First, within the MST [4,5], it is plausible to hypothesize that the chronic exposure to minority stress, from the very early years of life, might also favor the development of dysfunctional personality features and interpersonal patterns that mimic the symptoms of PDs. This might be particularly true for transgender people, as opposed to members of other sexual minorities, since their awareness of their experienced gender can appear at a very early age, together with gender non-conforming behaviors and expression, that put them at risk for stigmatization, bullying, harassment, and violence [27,28,29]. This could be particularly crucial for a country like Italy, where the current study was carried out [30,31,32,33,34]. According to *Transgender Europe* [35], Italy was the second European country in the overall number of homicides of transgender people perpetrated between 2008 and 2016. The negative attitude toward transgender people is largely influenced by the Catholic Church and the influence that it still exerts on politics and the Italian society at large, which have caused the country to fall behind with the recognition of fundamental civil rights for all sexual minorities [36].

Second, personality pathology might also influence the clinical manifestations of GD, as well as the gender transition process. Although PDs (like any other mental health issue) should never be considered an absolute contraindication for gender transition, hormones or surgery [8], it is reasonable to assume that the presence of any PDs may interfere with the adaptation to the transition process, with compliance with medical treatments and with the post-hormonal and surgical well-being and satisfaction. Bodlund and Kullgren [37], for example, found that any PD diagnosis and a high number of fulfilled Axis II pathological traits were associated with negative post-surgery outcome. More generally, people with PDs can be difficult medical patients, especially when the condition they are affected with requires long-term medical management (as it is the case with hormonal treatments in medicalized transgender people). For instance, borderline symptoms significantly predicted reduced treatment compliance with several aspects of general healthcare among participants in a primary care population (i.e., conscientiousness with medical treatment, regular dental check-ups, timely completion of laboratory work, following doctor’s exercise and nutrition instructions, and remembering to take medications) [38].

### 1.2. Dimensional Models of Personality Disorders

Another line of research has explored the personality functioning of transgender people by means of dimensional models of personality, mostly with inconsistent results. Bozkurt et al. [39], for example, used the *Eysenck Personality Questionnaire* [40] to assess three personality dimensions and found higher scores on neuroticism in transwomen compared to cisgender men. Another study [41] explored the personality profile of people with GD by means of the *Temperament and Character Inventory* [42], showing that transgender and cisgender people displayed a similar personality profile. Furthermore, the personality profile of transgender individuals resembled more that of the control sample matched by gender identity rather than by gender assigned at birth. 

Generally speaking, the results derived from this line of studies provide a fragmented picture because of the different personality measures adopted. Most of these assessment instruments are not directly compatible with a clinical evaluation based on DSM PD criteria, and are not informative in terms of treatment planning.

To our knowledge, although previous studies have already assessed personality disorders and symptomatology in transgender populations, no study has yet explored the personality profile of these populations according to the new conceptual framework for PDs introduced by the DSM-5 AMPD [10]. The model allows clinicians to diagnose seven personality disorders, which are all characterized by a moderate or greater impairment in personality functioning and one or more pathological personality traits. The latter are intended as the “tendency to feel, perceive, behave and think in relatively consistent ways across time and across situations” (p. 762) [10] and are grouped in five broad domains (negative affectivity, detachment, antagonism, disinhibition, and psychoticism). The multidimensional approach of pathological personality traits is empirically derived [43] and has been operationalized in the Personality Inventory for DSM-5 (PID-5) [10].

### 1.3. The Current Study

This study aims to gain a more fine-grained picture of the personality pathology patterns among medicalized transgender men and women by evaluating both the dimensional personality domains and facets proposed by the AMPD [10] and the categorical DSM-IV PD diagnoses. As we start from the assumption that transgender identities are not pathological per se, we evaluate personality pathology as an outcome of the minority stress and social oppression experienced by transgender individuals.

To this aim, we compared the PID-5 patterns of transgender women and transgender men with those of, respectively, cisgender women and men derived from a large normative sample from the community [44]. It is important to note that there are currently no norms for transgender or non-binary people for most personality measures, including the PID-5. This puts psychologists and researchers in the position of having to decide whether to use the gender they were assigned at birth or the gender they identify with as a reference. This choice comes with several implications, as it might lead to over- or underestimating levels of distress or psychopathological symptoms, thus contributing to harm an already vulnerable population [45,46]. However, the limited number of studies and evidence available seem to suggest that, when scoring psychological assessment tools, it may be more effective to categorize individuals as their identified gender [47]. 

We also evaluated the prevalence of DSM-IV categorical PD diagnoses in the transgender women and men subsamples. The two subsamples were compared on the overall prevalence of PDs and of any specific PD diagnosis. In line with previous studies [8,20], we expected to find no difference in the overall PD prevalence, and a predominance of Cluster B diagnoses.

## 2. Materials and Methods 

### 2.1. Participants and Procedures

Participants were recruited among those consecutively admitted at the gender clinic at Niguarda Ca’ Granda Hospital, Milan, Italy, between January 2017 and July 2018. All participants were referred to the gender clinic for consultation on gender identity issues and/or gender-affirming hormonal (feminizing or masculinizing treatment) and/or surgical treatments. The criteria for inclusion in the study were a diagnosis of GD (according to DSM-5) [10], and absence of any current major psychiatric disorders (psychotic disorders, neurocognitive disorders, and mental retardation). Inclusion criteria were assessed through at least three individual sessions with an experienced mental health professional.

One-hundred-eight transgender participants accepted to participate in the study, but the final sample included only 87 participants (40 transgender women and 47 transgender men), as 21 failed to complete the questionnaires in their entirety or dropped out after the first assessment session. 

Participants first completed a set of self-report questionnaires (including the PID-5 and the SCID-II-PQ), and they were then evaluated with a structured interview for the assessment of personality pathology (SCID-II). Sociodemographic characteristics of the sample are summarized in Table 1. All participants were Italian citizens and the large majority (85%) of them identified as binary. No age difference was found between the two samples, *t* = 0.02, *df* = 85, *p* > 0.05). Thirteen participants (15% of the whole sample) were already taking masculinizing or feminizing hormones, not necessarily under medical advice.

The study was approved by the Institutional Review Board of the University of Milano–Bicocca and was designed in accordance with the ethical standards of the Declaration of Helsinki. All participants provided written informed consent to participate in the study.

### 2.2. Measures

#### 2.2.1. Dimensional Personality Domains

The Personality Inventory for DSM-5 (PID-5) [44,48] is a self- report questionnaire composed of 220 items, each of which is scored on a four-point Likert scale, from “very false or often false” to “very true or often true.” The questionnaire measures the 25 traits proposed by the alternative model of the DSM-5, Section III. Higher scores reflect higher levels of endorsement of that specific trait and personality pathology. To compute the personality domains and facets score we made use of the algorithm proposed by Krueger et al. [49]. In the current study, Cronbach’s α values of 0.96, 0.95, 0.83, 0.89, and 0.94 were observed respectively for Negative Affectivity, Detachment, Antagonism, Disinhibition, and Psychoticism, respectively. As for the facets, Cronbach’s α was acceptable (i.e. > 0.70) for all traits scales and ranged from 0.72 (Restricted Affectivity; Deceitfulness) to 0.95 (Eccentricity), except for Suspiciousness (α = 0.64) and Irresponsibility (α = 0.61). 

#### 2.2.2. Categorical Personality Disorders

The Structured Clinical Interview Axis II for DSM-IV (SCID II) [50] is a semi-structured interview that allows a categorical assessment of the ten DSM-IV PDs, as well as of PD not otherwise specified and of the adjunctive diagnoses of Depressive and Passive-Aggressive PDs. The SCID-II is preceded by the administration of its self-report screening questionnaire.

### 2.3. Statistical Analyses

Participants (*N* = 21) with more than 25% of missing responses on PID-5 questionnaire were removed from the sample, according to the scoring norms [49].

We then compared the mean scores of each PID-5 facet and domain between transgender clients and the corresponding normative data derived for the Italian general population [51] using one-sample t tests. Transgender women (assigned male at birth) were compared with cisgender women, while transgender men (assigned female at birth) were compared with cisgender men.

Finally, we evaluated the prevalence of PDs in the overall sample, and then compared PD rates between transgender women and transgender men using χ^2^ test.

## 3. Results

### 3.1. PID-5 Profiles in the Study Sample

Table 2 shows PID-5 scores in the transgender women and transgender men samples and their comparison with those of cisgender women and men from the general population. 

Transgender women scored lower than cisgender women on two main domains: Negative Affectivity, *t* (39) = −3.94, *p* < 0.001, 95% CI [−0.43, −0.14], and Psychoticism,* t* (39) = −2.83, *p* < 0.01, 95% CI [−0.31, −0.05]. Specifically, they scored lower on two facets of Negative Affectivity, i.e., Submissiveness, *t* (39) = −4.96, *p* < 0.001, 95% CI [−0.51, −0.22], and Perseveration,* t* (39) = −3.35, *p* < 0.01, 95% CI [−0.43, −0.11], as well as on two facets of Psychoticism, i.e. Unusual beliefs, *t* (39) = −3.66, *p* < 0.001, 95% CI [−0.37, −0.11], and Cognitive and Perceptual Dysregulation, *t* (39) = −3.34, *p* < 0.01, 95% CI [−0.30, −0.07]. Further, transgender women scored lower than cisgender women on the Disinhibition facets of Impulsivity, *t* (39) = −3.80, *p* < 0.001, 95% CI [−0.52, −0.16].

As for transgender men, lower scores than cisgender men were found on Antagonism *t* (46) = −7.19, *p* < 0.001, 95% CI [-0.38, −0.21], and specifically on all the facets of this domain, i.e., Manipulativeness, *t* (46) = −4.74, *p* < 0.001, 95% CI [−0.46, −0.18], Deceitfulness, *t* (46) = −11.56, *p* < 0.001, 95% CI [−0.47, −0.33], Grandiosity, *t* (46) = −8.19, *p* < 0.001, 95% CI [−0.51, −0.31], and Callousness, *t* (46) = −8.38, *p* < 0.001, 95% CI [−0.40, −0.25]. Also, transgender men scored lower on the Negative Affectivity facet of Restricted affectivity, *t* (46) = −2.97, *p* < 0.01, 95% CI [−0.43, −0.08], on the Detachment facet of Intimacy avoidance, *t* (46) = −3.22, *p* < 0.01, 95% CI [−0.45, −0.10], and on the Disinhibition facets of Irresponsibility, *t* (46) = −4.68, *p* < 0.001, 95% CI [−0.41, −0.16], Impulsivity, *t* (46) = −2.87, *p* < 0.01, 95% CI [−0.51, −0.09], and Rigid perfectionism, *t* (46) = −2.81, *p* < 0.01, 95% CI [−0.44, −0.07]. 

### 3.2. DSM-IV Personality Disorders Rates in the Study Sample

SCID-II results are summarized in Table 3. Ninety-one participants (43 transgender men and 48 transgender women) completed the semi-structured interview out of the initial sample. Forty-five participants were diagnosed with at least one PD (49.5% of the total sample), and eight participants (8.8%) received more than one PD diagnoses. Cluster B PDs were the most frequent diagnoses, affecting 18.7% of participants in the overall sample. Cluster C PDs were diagnosed in five cases (5.5%) and Cluster A PDs in three cases (3.3%). Twenty-five participants (27.5%) received a diagnosis of not-otherwise specified (NOS) PD. The specific disorders with the highest prevalence rates were borderline PD (11%), depressive PD (although not included in the official classification) (9.9%), and narcissistic PD (5.5%). Narcissistic PD was more prevalent among transgender women than transgender men. No difference in the prevalence of any other PD was detected between the two subgroups of transgender participants.

Given the discrepancies between the self-report and clinical assessment of personality functioning, we further evaluated whether the presence of any DSM-IV PD diagnosis was associated with higher scores on the PID-5 domains by means of five ANOVAs with a 2 (affirmed gender) × 2 (PD vs. No PD) design (dependent variables: PID-5 domain scores).

Transgender participants with at least one PD diagnosis showed higher scores on Negative Affectivity, Detachment, Disinhibition, and Psychoticism (*Fs* (1, 79) between 9.288 and 17.371, all *ps* ≤ 0.003), while the presence of any PD diagnosis was unrelated with Antagonism scores, *F* (1, 79) = 1.101, *p* > 0.05. Further, transfeminine individuals showed higher scores than transmasculine individuals on Antagonism (significant main effect of affirmed gender: *F* (1, 79) = 5.944, *p* < 0.05).

## 4. Discussion

This study highlighted two main findings. First, the AMPD dimensional assessment of personality traits indicated that the transgender sample presented, overall, a healthier and less maladaptive personality profile than their cisgender counterparts matched by gender identity. Second, the categorical approach to PDs diagnoses by means of structured clinical interviewing pointed out a considerable prevalence of PDs in our sample: almost half of the included transgender clients exhibited at least one PD diagnosis.

Thus, two different scenarios emerged, with self-report dimensional measures offering a much healthier and less maladaptive view of overall personality functioning than the clinician-based categorical assessment. These different findings can be attributed to various sources. 

One hypothesis is that the differences might be connected to method variance. It has been reported that the variance of PD diagnoses explained by PID–5 traits is weak to moderate, ranging between 24% and 49% [52].

Another possible explanation is connected to the peculiarities of the therapeutic relationship and rapport between mental health professionals and transgender people requesting gender-affirming medical treatments. Transgender people may perceive the assessment process as a hurdle that must be cleared to achieve their goals, rather than as a useful and helpful clinical tool [20]. The clinician’s “gate-keeping” role may thus induce transgender people to present themselves (especially when confronted with self-report measures) as healthy and well- functioning, denying the presence and clinical relevance of overt psychopathological manifestations. Although current diagnostic manuals [10,53], clinical guidelines [54], and *Standards of Care* [9] moved far beyond the “gate-keeping” approach, even today, the fear of being denied appropriate treatment may result in transgender people experiencing mental health professionals as “gate keepers” in their transition journey [55]. As a consequence, they might show a guarded, defended attitude whenever questions or issues around their mental health are raised in clinical consultation, especially, but not only, when access to gender-confirming medical treatment is at stake. It must be noted, however, that in spite of this possible confounding effect, we were still able to discriminate between the dimensional personality profile of transgender people with and without any PD diagnosis, with transgender people with PDs showing a more dysfunctional and maladaptive profile than those without any personality diagnosis.

Also, it has been reported that clinicians, in the absence of solid TGNC affirmative training and awareness, may be biased in pathologizing transgender applicants for medical treatments. For instance, in a qualitative study with 45 TGNC participants, Mizock and Lundquist [56] identified specific missteps psychotherapists make in working with this vulnerable group, including gender pathologizing. Clinician bias against transgender clients has been also documented in experimental studies using case reports of fictitious patients [57,58]. It is possible that the discrepancies between self-rated and clinician-rated personality assessment in the present study may be at least partially attributed to some form of implicit bias in the clinicians. 

Finally, less maladaptive profiles might be read through the lens of the Minority Stress Model, specifically in terms of resilience. There is evidence, indeed, that transgender individuals use adaptive strategies to buffer the effects of stigma on health, exercising resilience to contrast societal stigma and promoting social adjustment [32,33,59]. Resilience in transgender people involves a self-generated definition of self and embraces self-worth [60], thus promoting wellbeing and health and representing a strong protective factor against mental disorders [33]. In the following two subsections, we will discuss in detail our findings. The impact of minority stress may vary according to the level of trans-negative attitudes in the social and cultural environment and is therefore a function of the sociopolitical milieu [61].

### 4.1. Personality Facets and Domains According to the AMPD

Transgender men scored lower than cisgender men on almost every trait included in the Antagonism domain. Antagonism, juxtaposed to Agreeableness, is defined as “behaviors that put the individual at odds with other people, including an exaggerated sense of self-importance and a concomitant expectation of special treatment, as well as a callous antipathy towards others, encompassing both unawareness of others’ needs and feelings and a readiness to use others in the service of self-enhancement” (p. 780) [10]. 

The Antagonism domain has been reported to show marked gender differences, with men scoring consistently higher than women [51,62]. Manipulativeness, Deceitfulness, Grandiosity, Callousness and Hostility, as facets included in the Antagonism domain, seem therefore to be less characteristic of the personality functioning of transgender men as opposed to cisgender men. One speculative explanation for this result could be ascribed to a lower propensity of transmen to a sense of entitlement and self- assertiveness, as they navigate a world in which they are more exposed to aggressions, microaggressions, harassment and violence than cisgender men [63,64]. Instead, they might need to be more vigilant and attuned to other people’s feelings and needs in order to understand their intentions and attitudes. The low scores in the Antagonism domain in the sample of transgender men are in line with the low prevalence, in this sample, of narcissistic PD, of which Antagonism is a crucial clinical correlate [10]. Moreover, it must be noted that transgender men lived part of their lives in a female role and were socialized as women. Women are socially expected to be more agreeable than men, and generally report higher levels of agreeableness across most cultures around the world [65]. Also, on average, females report to be more nurturing, warm, affiliative than males, as well as less aggressive, impulsive, dominant, sensation-seeking, and risk-taking [66]. Lower scores on the traits included in the Antagonism domain (together with lower scores on other traits like Restricted Affectivity, Intimacy Avoidance, Irresponsibility, and Impulsivity) could be ascribed to the fact that transmen were encouraged, during their early years, to express their feelings and build intimate relationships, as well as being empathetic, attuned to the needs of others, and altruistic.

The same explanation can apply to the finding that transgender women show lower scores than cisgender women on Negative Affectivity, and specifically on Submissiveness and Perseveration. Being socialized as male might lead transgender woman to exhibit a reduced tendency to experience “frequent and intense experiences of high levels of a wide range of negative emotions and their behavioral and interpersonal manifestations” (p. 779) [10]. Several studies have shown gender differences in emotion expressions in Western cultures [67]. In particular women show greater emotional expression overall [67,68] and a wider range of positive [69] and internalizing negative emotions [70]. It could be argued that a masculine socialization encourages the adoption of a pattern of emotional expression that is consistent with the stereotypical gender role (i.e., emotional restraint). Transmen scores on Depressivity were marginally significantly higher (*p* < 0.05) than cisgender men only, a facet denoting “feelings of being down, miserable and/or helpless; pessimism about the future; pervasive shame and/or guilt; feelings of inferior self-worth; thought of suicide and suicidal behavior” (p. 779) [10]. This result is also supported by the relatively high prevalence of Depressive PD (approaching 12%) in the sample of transgender men.

The validity of Depressive PD as a discrete diagnostic entity has been criticized on several grounds [71], including the large diagnostic overlap with mood disorder diagnoses, dysthymia in particular [72]. Such critiques have led to the exclusion of Depressive PD from the DSM-5 [10]. The high endorsement of Depressivity in transgender men suggests that attention should be paid to signs of depression in both the form of a full-blown mood disorder and also as a personality disposition. Our findings confirm previous studies that found high rates of depression in transgender populations [11,12,13,14]. However, the strength of the current paper consists in the use of measures that are more precise and specific than those used in earlier studies, which were often a combination of sociology and social psychology research. Notwithstanding, although several studies have underlined the role of minority stress in increasing the risk for mood disorders in sexual minorities, only a few focused specifically on the transgender population [5,73], and none on the impact of minority stress in shaping long-lasting, and stable depressive personality traits (i.e. pessimism, shame, low self-esteem, lack of motivation, guilt-proneness), which seem to be pervasively characteristic of transgender people [74].

### 4.2. Categorical Diagnoses of PDs

The nearly 50% prevalence rate of any PD diagnosis in the current sample is consistent with previous findings on a sample of transgender people with DSM-IV “gender identity disorder.” For example, in the study by Madeddu et al. [20], using the SCID-II, an overall prevalence of PDs of 52% was found, with 22% prevalence of Cluster B PDs (with narcissistic PD showing the highest prevalence in the overall sample), 12% of Cluster A PDs, 2% of Cluster C PDs, and a 16% prevalence of Not Otherwise Specified PD.

In line with previous studies [8,20], transgender women and men did not differ in terms of PD rates, and borderline PD was the most frequent diagnosis in the overall sample [26], again with no differences in prevalence between the two subsamples. As the prevalence of PDs in the general population has been estimated around 15% in large epidemiological studies [23], it may be argued that transgender people are at a higher risk of developing PDs than the general population. However, the complex relationship between the development of PDs and GD is still far from being elucidated, and several factors may contribute to the high PD rates detected among transgender samples, as compared to general norms. Although PDs and GD may well be independent conditions, it might be difficult to ascertain the presence and clinical relevance of PD traits during the assessment process with transgender clients. The diagnosis of PDs often requires a longer clinical observation than that of GD, and every personality trait or interpersonal pattern assessed in transgender people should be carefully considered in the light of the specific phase of the transition process people are going through. For instance, ideas of reference or suspiciousness might be quite common in transgender people, especially at the beginning of their social transition [23]. Furthermore, these personality traits and patterns can be even adaptive at early stages, given the risks and threats (in terms of violence and interpersonal rejection) connected to the exoticization and ridicule of transgender bodies within a trans-negative cultural environment [75]. 

Moreover, as the onset of PDs and often that of GD can be traced back to adolescence/early adulthood (and sometimes even childhood), it might be argued that PDs (or maladaptive personality traits) may evolve as a dysfunctional way of coping with GD [76]. For example, transgender people might handle intense dysphoric feelings through social isolation, avoidance of interactions with others, self-harm and self-mutilation, anger outbursts, preoccupation with grandiose fantasies, and/or envy of others, all of which constitute characteristic patterns of definite PDs.

Another possible explanation could be linked to the MST [3,4] according to which the high prevalence rate of PDs and maladaptive personality traits in people belonging to minorities might result from the discriminative and stigmatizing environment in which they were raised and live. Transgender and gender non-conforming people are more likely than cisgender people to experience physical, psychological, and sexual violence, discrimination, harassment, rejection from and friends and family [77,78], even the more so than people belonging to other sexual minorities (i.e., lesbian, gay, and bisexual (LGB) people). Such negative experiences can impact on transgender people from the very early years of life. Over time, such negative experiences of discrimination end up conveying to the transgender person the message that their identity and their core self are unacceptable, representing an unremitting form of invalidation. Although to our knowledge no previous study has explored the long-term impact of minority stress on personality traits and disorders, there is some preliminary evidence derived from studies carried out in large samples of LGB people. For example, in a study on teenagers from the community [79], non-heterosexual participants were found to score higher on measures of borderline PD features than their straight counterparts, and the results remained significant even after controlling for measures of depression and anxiety. The authors argued that the association between a non-heterosexual orientation and borderline PD features can be explained as the result of an emotionally invalidating environment which, according to some theoretical models of borderline PD, plays a central role in the development of borderline PD [80]. Children at risk for developing BPD later in life are assumed to be born with a biological predisposition toward strong emotional responses that, in an interaction with the environment, are more likely to be invalidated. Over time, chronic invalidation leads to self-invalidation, as the capacity to accept and therefore manage one’s own emotional, cognitive, and behavioral experiences and responses is disturbed. This pattern is thought to lead to the emotional dysregulation at the core of borderline PD. The same considerations can be extended to transgender people [81] to explain the high prevalence of borderline PD and traits assessed in the current study, as well as previous ones [26]. It must be noted however that the current formulation of PDs criteria in DSM-5 does not allow to exclude gender minority stress–related symptoms that resemble the pathological traits of any PD diagnosis. 

### 4.3. Limitations

Transgender people who seek medical treatments are not representative of the transgender and gender-nonconforming population at large, so the current results cannot be generalized to the entire transgender population. In fact, the prevalence of people in the general population identifying as gender-variant or gender-nonconforming is much larger than that referred to gender clinics [82].

Also, we sampled participants at different stages of treatment and social transition: some people included in the sample were already taking hormonal therapies (not necessarily under medical advice), some others were already living in their experienced gender, while some others could be at an earlier stage of social transition. The different stage of transition might have influenced the response patterns to self-administered tests and clinician-administered interviews.

Longitudinal studies are needed to clarify the impact of personality pathology on long-term outcomes of medicalized transgender people. To our knowledge, this relationship was only evaluated with respect to satisfaction with gender-affirming surgery [37]. More studies are necessary to ascertain whether, in this specific population, the presence of any PDs traits or diagnosis (and borderline PD in particular) [38] can interfere with compliance with medical treatments and lead to worse general health outcomes (i.e., regular endocrinological check-ups, laboratory testing, following doctor’s instructions).

Finally, in the current study, we compared the dimensional personality profile of transgender men and women with cisgender controls matched by gender identity. The lack of a consistent method for categorizing the gender of persons who identify differently than the gender they were assigned at birth presents a significant limitation to researchers attempting to study gender minority populations [49]. It is advisable that improvements in deriving assessment norms are made due to the growing visibility and acceptance of transgender people in the general public.

## 5. Conclusions

Our results call for the importance of an accurate assessment of personality pathology and maladaptive traits in transgender clients referred to gender clinics, by means of both categorical and dimensional assessment tools, in order to tailor the gender-affirming treatment to the client’s specific needs and vulnerabilities. It is deserved, once again, to highlight that such an assessment of personality pathology must not be discerned from the evaluation of the more general psycho-social condition in which transgender persons live and, thus, from the minority stressors that might have influenced their mental health.

Also, the presence of specific vulnerabilities in terms of personality functioning might be useful to anticipate potential difficulties that might be experienced in the following phases of the transition, especially, but not only, in the interpersonal and social domains. The presence of a full-blown PD diagnosis should be addressed with ad-hoc treatments in order to improve the client’s overall functioning.

## Figures and Tables

**Table 1:** Demographic characteristics of the sample (*N* = 87).

Demographics	Transwomen (*n* = 40) *n* (%)	Transmen (*n* = 47) *n* (%)
Age	28.21 (SD = 11.8)	28.26 (SD = 11.6)
17	2 (5%)	2 (4.3%)
18–25	20 (50%)	26 (55.3%)
26–35	6 (15%)	7 (14.9%)
36–45	8 (15%)	6 (12.8%)
46–55	2 (5%)	4 (8.5%)
56–65	1 (2.5%)	2 (4.3%)
Non-binary identity	9 (22.5%)	4 (8.5%)
Hormone treatment	10 (25%)	3 (6.4%)
Educational level		
Less than high school	7 (17.5%)	17 (36.2%)
High school diploma	24 (60%)	21 (44.7%)
Master’s degree	7 (17.5%)	8 (17%)
Other	1 (2.5%)	1 (2.1%)
Civil status		
Single	31 (77.5%)	39 (83%)
Married	0	1 (2.1%)
Divorced	4 (10%)	1 (2.1%)
Cohabitant	4 (10%)	5 (10.6%)

**Table 2:** PID-5 descriptive statistics for PID-5 domains and facets and comparisons between transgender and cisgender women and transgender and cisgender men.

PID-5 Domains and Facets	Transmen (TM) (*n* = 47)	Cisgender Men (CisM) ^1^	*t*	Statistical Comparison	Transwomen (TW) (*n* = 40)	Cisgender Women (CisW) ^1^	*t*	Statistical Comparison
	Mean (SD)	Mean			Mean (SD)	Mean		
Negative Affectivity	0.85 (0.52)	0.93	−1.11		0.75 (0.46)	1.04	−3.94 **	CisW > TW
Emotional Lability	1.12 (0.88)	0.97	1.16		0.98 (0.70)	1.17	−1.73	
Anxiousness	1.17 (0.71)	1.04	1.32		1.01 (0.60)	1.14	−1.30	
Separation Insecurity	0.77 (0.77)	0.80	−0.25		0.75 (0.67)	0.82	−0.66	
Submissiveness	0.54 (0.63)	0.70	−1.77		0.34 (0.47)	0.71	−4.96 **	CisW > TW
Hostility	0.81 (0.69)	0.97	−1.69		0.82 (0.62)	0.90	−0.86	
Perseveration	0.81 (0.52)	0.94	−1.74		0.69 (0.51)	0.96	−3.35 *	CisW > TW
Restricted Affectivity	0.73 (0.59)	0.99	−2.97 *	CisM > TM	0.69 (0.53)	0.80	−1.26	
Detachment	0.86 (0.48)	0.85	0.25		0.75 (0.44)	0.78	−0.54	
Withdrawal	0.96 (0.63)	0.79	1.90		0.74 (0.61)	0.69	0.56	
Intimacy Avoidance	0.54 (0.59)	0.82	−3.22 *	CisM > TM	0.66 (0.69)	0.78	−1.11	
Anhedonia	1.01 (0.64)	0.93	0.81		0.82 (0.60)	0.89	−0.67	
Depressivity	0.79 (0.68)	0.56	2.28	CisM < TM	0.53 (0.52)	0.53	0.09	
Suspiciousness	1.01 (0.54)	1.05	−1.07		0.98 (0.41)	0.99	−0.23	
Antagonism	0.37 (0.28)	0.67	−7.19 **	CisM > TM	0.58 (0.44)	0.55	0.37	
Manipulativeness	0.33 (0.47)	0.65	−4.74 **	CisM > TM	0.61 (0.56)	0.55	0.68	
Deceitfulness	0.28 (0.24)	0.68	−11.56 **	CisM > TM	0.40 (0.39)	0.55	−2.52	
Grandiosity	0.26 (0.35)	0.67	−8.19 **	CisM > TM	0.50 (0.54)	0.55	−0.67	
Attention Seeking	0.70 (0.57)	0.90	−2.42		1.01 (0.78)	0.84	1.38	
Callousness	0.28 (0.27)	0.61	−8.38 **	CisM > TM	0.37 (0.36)	0.43	−1.04	
Disinhibition	0.77 (0.40)	0.87	−1.82		0.76 (0.36)	0.82	−0.98	
Irresponsibility	0.43 (0.42)	0.72	−4.68 **	CisM > TM	0.51 (0.35)	0.62	−1.98	
Impulsivity	0.75 (0.72)	1.05	−2.87 *	CisM > TM	0.70 (0.57)	1.05	−3.80 **	CisW > TW
Distractibility	0.72 (0.65)	0.85	−1.44		0.65 (0.63)	0.79	−1.33	
Risk Taking	1.05 (0.48)	1.10	−0.69		0.90 (0.42)	1.02	−1.79	
Rigid Perfectionism	0.89 (0.63)	1.15	−2.81 *	CisM > TM	1.05 (0.51)	1.12	−0.85	
Psychoticism	0.57 (0.53)	0.67	−1.23		0.46 (0.40)	0.64	−2.83 *	CisW > TW
Unusual Beliefs and Experiences	0.50 (0.52)	0.61	−1.44		0.39 (0.41)	0.63	−3.66 **	CisW > TW
Eccentricity	0.75 (0.79)	0.86	−0.98		0.64 (0.63)	0.76	−1.22	
Cognitive and Perceptual Disregulation	0.47 (0.50)	0.53	−0.88		0.34 (0.35)	0.53	−3.34 *	CisW > TW

^1^ Data derived from the normative sample of the validation study of Italian version of the PID-5 [51]. * *p* < 0.01; ** *p* < 0.001.

**Table 3:** Prevalence of personality disorders in the two samples.

Personality Disorders	Transwomen (*n* = 48)	Transmen (*n* = 43)	Total (*N* = 91)
At least one PD diagnosis	26 (54.2%)	19 (44.2%)	45 (49.5%)
Cluster A diagnoses ^1^	1 (2.1%)	2 (4.6%)	3 (3.3%)
Paranoid PD	1 (2.1%)	0	1 (1.1%)
Schizoid PD	0	1 (2.3%)	1 (1.1%)
Schizotypal PD	0	1 (2.3%)	1 (1.1%)
Cluster B diagnoses ^1^	8 (16.7%)	4 (9.3%)	12 (13.2%)
Histrionic PD	1 (2.1%)	0	1 (1.1%)
Narcissistic PD	5 (10.4%)	0	5 (5.5%)
Borderline PD	6 (12.5%)	4 (9.3%)	10 (11%)
Antisocial PD	0	1 (2.3%)	1 (1.1%)
Cluster C diagnoses ^1^	2 (4.2%)	2 (4.6%)	4 (4.4%)
Avoidant PD	1 (2.1%)	1 (2.3%)	2 (2.2%)
Dependent PD	1 (2.1%)	0	1 (1.1%)
Obsessive-Compulsive PD	1 (2.1%)	1 (2.3%)	2 (2.2%)
Not-otherwise specified (NOS) PD 2	16 (33.3%)	9 (20.9%)	25 (27.5%)
Depressive PD	4 (8.3%)	5 (11.6%)	9 (9.9%)
Passive-Aggressive PD	0	0	0

^1^ At least one PD diagnosis belonging to this cluster. ^2^ We included here only those individuals who fulfill the general diagnostic criteria for a PD but that do not meet criteria for any specific PD. The prevalence of the PDs not officially included in the classification (depressive and passive-aggressive) are listed separately.
